# Inflammation: Its Symptoms, Causes, and Treatment Philosophically Considered

**Published:** 1850-02

**Authors:** 


### BIBLIOGRAPHICAL NOTICES.

*Inflammation: its Symptoms, Causes, and Treatment philosophically considered.* By J. P. Batchelder, M. D. New York. 1848. Pamphlet, 8vo. pp. 66.

The object of this essay, and the motives which induced its author to prepare and publish it, are thus unfolded by himself: "So much has been written on inflammation, that it may be deemed quite superfluous to present to the medical public any additional remarks; but having seen no pathological views from which the indications of cure, and the methodus medendi seemed to flow as natural, regular and legitimate deductions, the writer proposes for consideration a theory which he hopes may contribute to remedy that defect." And he adds, "Whether the endeavor will be crowned with success, or the views presented meet with any favor, other than a candid examination, is to him quite problematical." p. 3.

We will endeavor to give a brief synopsis of the author's views.

He considers that the *proximate* cause of inflammation—that phenomenon, in the language of Parry—" that phenomenon in the body or part most immediately preceding the state which we call disease, and without which previous phenomenon the disease is not known to exist," is " a suspension of the normal resistance, a relaxation, a yielding, a diminished action of the capillaries of the part that admits, upon hydraulic principles, of the inflowing of fluids by which they (the capillaries) are over distended. This want of action, this non-resistance, continues until the distention has proceeded so far as to excite actual resistance in the capillaries, and then, and not until then, can we affirm the existence of the disease. The action of the vessels is then increased. This increased action" (*contraction*, the only mode of increased action of which the capillaries are capable, as stated at page 16,) " the final end and natural consequence of which is, not only to prevent the additional influx of fluids, but to force them out of the inflamed part, constitutes the true *vis medicatrix naturæ;* for it causes, or attempts to cause, and if successful does cause, the reflux of fluids into the general circulation, which is equivalent to an evacuation by art." p. 17.

Thus, it will be perceived, inflammation is supposed by the author to consist, at its outset, in a painful distention of the capillaries, a condition of commencing, active, salutary resistance to a preceding state of relaxation, its proximate cause.

The occurrence of this primary and essential state of relaxation is thus explained: The blood which circulates in the capillaries is regarded as their natural stimulus which prompts them to action or resistance. When the vessels are healthy, and their contents natural in quantity and quality, action and reaction are duly balanced, and every function normally performed. And before this equipoise can be subverted, and an anormal relaxation of the capillaries induced, the author contends that a stronger impression must be and is exerted upon a part, than that made by the fluids on the inner surfaces of their containing vessels; in which event, and in obedience to the axiom that the stronger effaces the weaker impression, the vessels " cease their resistance, and yielding, suffer themselves to be distended, over-distended, and stretched, until they are excited to resist, and that painfully," p. 14. The agents by which this state of things is brought about constitute the *exciting causes* of inflammation. They may be " external agents acting mechanically, chemically, or physically upon any part of the body; or causes affecting the whole system; or the disease may arise spontaneously,—that is, without any apparent cause." The first-mentioned class of causes operates directly by producing a stronger impression on the surface upon which they act, than that made by the blood upon the internal surface of the capillaries beneath this surface, and consequently and indirectly occasions a relaxation of these vessels; and since, naturally, the blood in the capillaries is acted upon all over the system, at the same instant and with an equable force, (the *vis a tergo*,) if the resistance opposed to this force is weaker at one point than elsewhere, thither will there be a greater afflux of blood. The *second* class, among which the author considers cold and malaria, is supposed to "excite a general contraction of the capillaries throughout the entire body, which is followed by a spontaneous relaxation commensurate in extent and degree with the previous contraction ; if under such circumstances any part or organ, from predisposition, hereditary taint, disease, injury, age or sex, be weaker than the rest of the system, its vessels having less power of resistance, will yield and be over-extended, an effect which will be greatly enhanced by the increased energy with which the heart will act in consequence of its own capillaries having, simultaneously with those of the other organs, relaxed, and of course admitted more arterial blood into its very textures, as well as into its cavities." Or, if the cause thus acting be extensive and powerful, and applied to the surface, the vessels of some internal organ, in obedience to the law propounded, may relax and suffer themselves to be over-distended to the development of inflammation. p. 15. *Thirdly*, there may be no apparent cause, and yet in consequence of "great weakness, or readiness in the capillaries of some part to relax and yield to the distending influence of the fluid which they contain," the same phenomenon may be induced. p. 15.

The author easily explains the symptoms which are considered as denotive of inflammation,—" redness, swelling, pain and heat." "1st. Redness arises from an increased determination and admission of blood into the vessels of an inflamed part, and particularly such as in a state of health circulate colorless fluids." He regards this as an essential symptom, and he thinks that, although it sometimes exists without inflammation, the latter cannot exist when it is absent ; and further, that by the degree of the redness present, the stage, rapidity and violence of the disease may be pretty accurately distinguished. p. 3. 2d. The tumefaction or swelling he ascribes to two causes,—to the presence in the vessels of an unusual amount of blood, " proportioned to the diminution of their resistance and the intensity of the *vis a tergo ;* and also, and chiefly, to the effusion of serum, albumen and coagulable lymph." p. 4. Concerning this latter cause of the swelling, the author thus reasons. " How does this effusion take place, and by what law is it governed or regulated ? We come to the principle. If from any cause the capillaries be suddenly or violently distended, or over-distended, and consequently prompted to act violently, or inordinately, as in inflammation, the pores, like the sphincter of the bladder, or of the anus, similarly circumstanced, act or resist no less violently or inordinately, and consequently suffer no fluid to escape; and this we conceive to be the precise state of the vessels and pores in the early stage of that disease, and is analogous to that of the vessels on the surface during the hot stage of an intermittent, in which the skin is dry.

" At this period there is no other swelling than that occasioned by the distended state of the vessels of the part. There is no effusion of serum, none of fibrin. There is mere congestion; the vessels over-distended, the pores constricted and resistant. How then, we repeat, is the effusion produced? The principle is this, that rest shall succeed to action. This law results from necessity, and is consequently universal, reaching and controlling all vital movements; hence, vascular contraction is followed by the spontaneous relaxation to which we have alluded, and which, generally, is in proportion to the previous contraction. Therefore, in obedience to this law, the pore, or sphincter, as the case may be, which has been for some time resisting powerfully and completely the inordinate action of the vessel or viscus, and of course preventing the transmission of the fluids by which the latter (the vessel or viscus) were distended, now in its turn foregoes its resistance, relaxes and allows the effusion of serum or fibrin to take place into the inflamed part, or perspirable matter over the whole surface, as in the sweating stage of an intermittent, (or the escape of its contained fluid from the containing viscus, Rev.) In these cases the overdistended vessels are relieved. The final end of this effusion is to empty the vessels; in other words, to cure the disease. This is the method of nature, and also of art—a fact of which the practitioner should never lose sight.

" If this want of equilibrium between the vessels and pores continues but for a short time, and the effusion be but slight, resolution is said to have taken place, which is one of the terminations of inflammation mentioned by systematic writers; but with all due deference, we are disposed to consider the effusion by which the vessels are emptied and relieved as the only legitimate termination of that disease—if indeed we except gangrene. If the state of distention continue several days, the effusion may be considerable and of a mixed character, as serum, albumen, coagulable lymph, or even blood. It constitutes the principal cause of the swelling. We are, however, ready to admit that want of absorption and the generation of new vessels tend in some degree to augment the tumefaction; and here it is proper to remark that the formation of new vessels is often exceedingly rapid, which, with the secretion of pus, has been reckoned among the causes of swelling." pp. 5, 6.

The *third* symptom, *Pain*, the author says, "in all cases, without a single exception, as we believe, arises from one of two causes—an over-constricted, or an over-distended state of the vessels." And again, "it may be laid down as an axiom in physiology and pathology, that whatever diminishes the influx of red blood into a part, or forces it out, produces a corresponding diminution of its susceptibility of impression." p. 8. And the reverse, he also contends, is true—that the sensibility of a part is proportionally exalted by increments in the amount of red blood sent to it. The author's remarks upon the true signification of the pain suffered, and the specific character of pain which appertains to each particular tissue, are, we think, very just, generally.

The *fourth* symptom, *increased heat*, he does not regard as essential, or as particularly important. The inference drawn by him, from his premises and reasoning, is, "that this fourth symptom of inflammation is seldom present unless the skin, or mucous membrane in its immediate vicinity, is involved, and that it always indicates an over-distended state of the capillaries of the part, and gives intimation that changes are going on in the tegumentary structures, which interfere with their functions, or threaten their integrity." pp. 12, 13.

The plan of treatment recommended by the author does not differ, generally, from that pursued by physicians of judgment and experience. This branch of the subject is examined under two main points of view:—1st, the conditions upon which the over-distention of the capillaries are maintained; 2d, the mode of relieving this over-distention. The *first* depends upon the constant antagonism, as the author supposes, which exists between the *vis a tergo* and the "*pores*" of the capillaries, and the morbid predominance of resistance of the latter over the former, so that the fluid in the vessels is not permitted to escape. The *second*—

the method of obviating this condition—consists in lessening "the *vis a tergo* so as to restore the equilibrium between the vessels and the pores;" in diminishing "the effect of the fluids impinging against the inner surfaces of the capillaries, which excites them to inordinate action, and disturbs the harmony of function between the vessels and the pores;" and lastly, in counteracting the results of the disease. To secure these ends the whole antiphlogistic regimen and treatment is advised in full force—comprising strict attention to diet and hygienic conditions, general and local bleeding, the free use of emetics and cathartics, of arterial and nervous sedatives, the ordinary topical applications, and rest of the whole body, or of the affected part, in the proper position.

After having thus discussed the subject of inflammation, and its treatment, the author devotes the remaining pages, 47—66, of his pamphlet to the consideration of the commonly-called "*terminations*" of the disease, viz., Resolution, Adhesion, Suppuration and Ulceration—he himself, however, admitting of "but one legitimate termination, *i. e.*, by depletion," by which he means the removal of the fluids from the vessels of the inflamed parts, either by their spontaneous contraction, or by the assistance of art. pp. 17, 47.

It would be foreign to our purpose, at present, to enter into a discussion of the subject of inflammation, further than the bearing of this pamphlet upon it requires. We have given the author's tract a careful perusal, and have derived pleasure from it; but we think that it is amenable to some strong objections, and we shall state them in that spirit of candor and respectful consideration which the author himself invites.

It is well known that there are now two chief theories with regard to the condition of vital action in the capillaries of an inflamed part. Both admitting that these vessels are increased in diameter, in order to accommodate the augmented supply of blood which they are acknowledged to contain, one theory supposes that this dilatation is the result of an increased action of the capillaries, under the influence of the organic nerves, while the other contends that these vessels are in a debilitated condition, and that their enlargement is the result of a passive relaxation. The author of the treatise under examination adopts the latter view. He assumes, without examination, that the only mode in which increased action can be manifested in a capillary is a *diminution* of its calibre, and that, consequently, the first theory must be wrong. Without attempting to decide positively between these two views, we incline to adopt the first, believing that it is based upon more tenable ground, and that it affords a more easy and rational explanation of the phenomena of inflammation. We think that increased action of the capillaries, the result of increased organic nervous influence, is not only compatible with, but productive of expansion of these tubes; the phenomena of blushing and other local determinations of blood, under similar causes, and the condition of the capillaries in hyper-nutrition, render it more than probable that a similar condition of the vessels exists in inflammation. This active expansion may continue, or it may, at length, be changed, from exhaustion of nervous force, into a state of passive dilatation. This seems to us a more probable supposition, and more analogous to what we generally see of the effect of long-continued exercise of all organs, especially of hollow organs, than that the capillaries should, after long resistance to the distending power of the affluent blood, and after being " distended, and over-distended " thereby, be capable of active, sustained contraction so as to expel the blood from their interior. (p. 17.) And it renders unnecessary the assistance of such an influence as that which Dr. Batchelder invokes in the explanation of the occurrence of distention of the capillaries,—the action of an impression upon some part stronger than that made by the blood upon the inner surface of these canals. (p. 14.) The idea of the existence of such an influence or action is entirely groundless; for it supposes, of necessity, that the circulation of the blood in the capillaries is assisted by the elastic resistance of these vessels to the blood, " which is their natural stimulus, and prompts them to action." Now the most enlightened physiologists of the present day contend, that the contraction of the heart and the elasticity of the arteries are the forces which propel the blood through the capillaries, and that the latter exert no mechanical action upon it; that the motion of the fluid is equable, not pulsatory, as would be the case if the capillaries reacted upon the blood which filled them.

The capillaries possess a degree of *elasticity*, and a capability of accommodating their calibre to the amount of fluid which they contain; but this is a very different property from the *contractility* which responds to a stimulus, as that of the heart, which causes it to act upon and expel the blood from its cavities.

We dissent, also, from the author's views with respect to the structure of the capillaries. He regards them as being possessed of "*pores*," which, if we were to be guided by his mode of speaking and reasoning concerning them, are distinct and visible outlets. The walls of the capillaries partake, undoubtedly, of the general porosity of all membranous textures; but their pores are not visible, and still less are they, by any stretch of fancy, to be assimilated or likened to the sphinctered orifices of the bladder, uterus, rectum, and stomach, as is attempted by the author; (p. 19, et seq.) consequently, all the reasoning based upon this falsely assumed similarity of structure and function must pass for nothing.

The author has entirely neglected to notice the affinity which inflammation excites between the walls of the capillaries and the modified fluid which they contain, and the effect which this must have upon the circulation in the inflamed part.

The account of the symptoms of inflammation embraces no mention of altered functions of the tissue or organ involved, and the explanation of the symptoms is insufficient and faulty. Thus the pain of the disease is ascribed to stretching of the nervous fibrils, in consequence of the swelling of the part, and to over-distention or over-contraction of the capillaries, and is said to be closely proportioned to the amount of red blood circulating in them. The vital changes, of which the nerves of the part are the subjects, are not alluded to. The temperature, too, of an inflamed part is affected by other circumstances than the amount of arterial blood, and does often rise higher than that of the blood in the heart, in consequence of increased vital action occurring in the diseased structure itself. (Copland, and others.)

To the views expressed as to the treatment of inflammation, we have nothing particularly to object, excepting that they are chiefly or wholly applicable to the sthenic form of the affection; indeed, throughout the Essay, asthenic inflammation is very imperfectly, if at all, considered. In speaking of general bloodletting the author alludes to *syncope*, which, he says, " consists in a universal and extreme contraction of the capillaries;" and that he considers this to be an *active* condition, is obvious from the following sentence: "Now this violent and universal contraction of the capillaries must be followed by a *spontaneous relaxation,* or the patient will die." (pp. 25, 26.) How there can be such an active force in a condition characterised by such complete prostration of all energy as is syncope, we do not comprehend. Nor do we understand how the author can promulgate such an idea as this: "for an obvious reason we should be in no hurry to remove the syncope; as a general rule, the longer it continues, and the more slowly it goes off, the better." (p. 33.) We should rather inculcate the necessity of a speedy restoration, under the impression that the longer the syncope continued the greater the danger that it would be irrecoverable, and that, (if it be permitted us to perpetrate "a bull,") if the patient should recover, it would only be to find himself—*dead.*

The author's remarks concerning the organization of effused plastic matter, the mode of formation of new vessels, and the object of their formation, appear to us to be very erroneous. He says, p. 6, "the effused fluids, if too great in quantity to be absorbed, will remain an incumberance, greatly interfering with, or wholly suspending, the function of the organ; therefore, to get rid of them, the suppurative process is instituted by nature in the simplest manner possible; to wit, by the formation of new vessels and consequent organization of the effused lymph. The secretion of pus, then, is the final end of the formation of new vessels. How are they formed? On this point we shall hazard a conjecture, which, like the theory promulgated in this paper, was framed many years ago:—that a globule of blood escapes from a vessel, probably the same which furnished the lymph, through which it (the globule) makes its way, and thus forms a channel along which other globules, following close and in rapid succession, continue to move, until the track becomes a vessel; similarly occurring results take place on every side; globules after globules escaping from other vessels, pass in all directions, till channel runs into channel, and globule joins globule, when they will move in that direction in which they are most forcibly impelled by the *vis a tergo* which continues to urge them along, until the channels are converted into living vessels, temporary or permanent, according to subsequent circumstances."

We do not wish to deny the author's claim to originality of theory, as regards his explanation of the manner in which new vessels are formed in the effused fibrin, although we have before seen the same view expressed by other writers. We cannot, however, think that it is correct. A globule of blood can only escape from a capillary vessel through a rupture of the wall of the latter; and, even admitting that such a rupture does occur, and that a globule is forced by the *vis a tergo* through the rent, across the coagulated fibrin, which seems to us improbable, how is it supposed to find its way to a venous channel? We grant that the blood globule is a living cell, endowed with wonderful properties, physical and vital, and that nature is exhaustless in her resources and in her means of accomplishing her ends; but we can scarcely think that she would expend her energies in the mode advocated by the author. Let the reader imagine the curious and amusing spectacle presented by an indefinite number of ejected blood-discs careering "hither and yon," across an intervening septum of plasma, in search of refuge and shelter upon the opposite side; it will remind him of some of Milton's Wanderers in Chaos,—

> "Embryos and Idiots, Eremites and Friars
> White, black and grey, with all their trumpery."

We would rather embrace the views of Schwann, Vogel, Carpenter and some others, which suppose that vessels are formed in exuded fibrin in the same manner as in the original tissues in primary development; yet even here we acknowledge the difficulty of explaining the mode by which the new vessels unite and innosculate with those of the pre-existing surface or surfaces. Or we would still rather adopt the explanation of Mr. Kiernan and Mr. Travers, formed from observing that "inflamed capillaries become varicose, and at points project in pouches and diverticula, and stretch into loops. If these give way, argues Dr. Williams, the blood would be injected into the lymph; and if something of channels were previously formed by the arrangement of the fibrils of the latter, or the elongation and communication of cells, it is quite conceivable that a current would be effected by the *vis a tergo* through several openings, and that a return of the blood would take place by a reversal of the weaker currents." (*Williams' Princ.*, p. 252.) But no such rupture is necessary,—the blood being conveyed through channels formed by "out-growths" from the original vessels.—(*Travers*—Inflam. and the Healing Process; also, *Paget,* Sect. 4th, *London Med. Gaz.*—July 13th, 1849.)

However formed, "the final end" of their production is, we conceive, the organization of the effused fibrin, not "the secretion of pus," as Dr. Batchelder contends.

With reference to this latter point he says, p. 52, "Suppuration has been usually considered one of the terminations of inflammation; but the intermediate links between inflammation and suppuration seem to have been overlooked, viz., the effusion of coagulable lymph, and the formation of new vessels, which, with a single exception, (in the inflammation of mucous membranes, and this only an apparent exception,) always precede the secretion of pus. These new vessels which secrete the pus, with the parenchyma which connects them together, compose the granulations, which, being piled one upon another, fill the cavity, and constitute what is technically called "*union by the second intention.*" Although we sometimes speak of "laudable pus," the formation of matter is never the product of healthy action. It may be modified by the part affected, the state of the constitution, the cause and character of the disease." And again, (p. 53, note,) "we remarked that, if the effusion of coagulable lymph was so large in quantity as to prevent the new, or primitive, vessels, which shoot into it, from innosculating with one another, they immediately commence secreting pus, which is deemed to be "altered blood;" and that the alteration of the blood, so as to constitute it pus, is the function of the new vessels, but they answer a secondary process, viz., the organization of the lymph;—their peculiar, appropriate and primary function is, we repeat, the secretion of pus. All that is required is, an investment of fibrin about new vessels, or the old ones elongated, in order to enable them to perform this function." Again, (p. 7,) "the primitive vessels, if elongated into the adventitious lymph, will secrete pus. But the business, so far as an abscess is concerned, is probably done chiefly by the newly-formed vessels, which are little other than imperfectly animalized tubes, that can exert no other than a mere altering influence upon the blood, which it converts into pus."

We think that there are many inaccuracies in these and other passages, in which the author expresses his views on the nature of pus, and the mode of its formation. In the first place, we object to the idea that the blood-vessels perform the part of secreting organs under any circumstances; they have a single office, that of conveying blood, and hence are, at best, merely *accessories* to the process of suppuration, as of nutrition and secretion; these complex actions are the results of the operation of agents and influences external to the vessels upon the fluid which the latter have conveyed to the tissues—the centres of nutritive and secretory life. Neither can pus be considered as "*altered blood.*" It is the result of a special organization of exuded fibrin, " an organization on which the character of pus is dependent, and which distinguishes it from other morbid products." (*Vogel.*) It is essential to the formation of pus, says this distinguished pathologist, first, that a fluid shall be secreted or separated from the blood, to serve as a cytoblastema, and this fluid is fibrin, as it exists in the liquor sanguinis, or in coagulated blood; this plasma may be of a healthy character, or more or less abnormal, it may be fluid or solid; secondly, it is necessary that the pus-corpuscles be formed in and from this pabulum, owing, probably, to a tendency possessed by the exudation itself to develop organized products. " The uses of the formation of pus to the organism consist in this, that by its means exudations which were originally fluid, and would have become solid, are prevented from coagulating ; and those already coagulated again become fluid, and thus the conditions requisite for their removal are effected."—(See also the fourth lecture of Mr. Paget.) F. W. S.

